# Does Reflection on Everyday Events Enhance Meaning in Life and Well-Being among Emerging Adults? Self-Efficacy as Mediator between Meaning in Life and Well-Being

**DOI:** 10.3390/ijerph18189714

**Published:** 2021-09-15

**Authors:** Natalia Czyżowska, Ewa Gurba

**Affiliations:** 1 Institute of Psychology, Pedagogical University of Kraków, 30-084 Kraków, Poland; 2 Department of Philosophy, The Pontifical University of John Paul II, 31-002 Kraków, Poland

**Keywords:** meaning in life, psychological well-being, self-efficacy, intervention, mental health

## Abstract

In recent years, the issue of the meaning in life has aroused particularly great interest in researchers considering the question of whether and how, using simple interventions, outside the therapeutic office, the sense of meaning in life and well-being can be strengthened. The aim of this study was to explore whether interventions based on reflection on everyday, stressful situations can contribute to fostering the sense of meaning in life and psychological well-being among emerging adults. Additionally, we aimed to explore relationships between the above-mentioned constructs and self-efficacy. The research focuses on emerging adults, who, as statistics show, are the most vulnerable among all adults to various mental problems. A pretest–posttest control group design was used. The study involved 80 emerging adults (56 women and 24 men) who were randomly assigned to the experimental group, which completed specially prepared diaries for a week, or the control group. Participants completed the Meaning in Life Questionnaire, the Generalised Self-Efficacy Scale, and the Ryff Scales of Psychological Well-Being twice. In the experimental group, significant differences were noted between pretest and posttest in psychological well-being, especially in the area of relationships with others (M_pretest_ = 59.3; M_posttest_ = 65.07; t(39) = −11.40; *p* = 0.001) and purpose in life (M_pretest_ = 54.85; M_posttest_ = 58.21; t(39) = −3.15; *p* = 0.003), as well as self-efficacy (M_pretest_ = 28.06; M_posttest_ = 29.60; t(39) = −2.82; *p* = 0.007). There were no differences in the level of meaning in life. The analysis carried out showed that self-efficacy mediates the relationship between presence of meaning in life and psychological well-being (the Aroian test: z = 4.48; SE = 0.11; *p* = 0.0007).

## 1. Introduction

It is known that dealing with traumatic events may have positive psychological consequences for the functioning of the individual [1,2,3]. It is assumed that experiencing trauma undermines or threatens the existing core beliefs about oneself and about the world, leading to cognitive activation and a subsequent search for new meanings in one’s life [4,5,6]. Sense of meaning in life can be defined in many ways, however, most definitions draw attention to having a sense of purpose, understanding your own life and perceiving it as significant/meaningful [7,8]. Steger [9] mentions two main dimensions of meaning in life: purpose (an individual has life aspirations that are consistent with each other and motivate him to act) and comprehension (an individual is able to make sense and understand himself, the world, and the relationship between him and the world he lives in). There are, however, researchers who also point to the existence of three dimensions of this construct: purpose, significance (to what extent a person believes his/her life is important and meaningful), and coherence (a certain level of predictability that allows a person to make sense of his/her life) [10]. Although the indicated dimensions of meaning in life are not identical, the research results suggest that they are located lower in the hierarchy than the “global” sense of meaning, which is at the top [11]. While there has been a lot of research on posttraumatic growth and possible positive consequences of traumatic events in recent years [12,13], there are little data on daily negative events and their potential impact on meaning in life. Focusing on everyday experiences seems to be particularly important because, as the research results show, meaning in life comes more from mundane events than from the belief that there is some transcendent purpose [14]. However, it is worth noting that meaning in daily life is positively correlated with global meaning in life [15]. Researchers suggest that daily negative life events may cause a person to start making efforts to restore meaning. The results of experimental studies show that, in situations that threaten meaning, the respondents declared a higher level of sense of meaning in life as a compensatory maneuver [16]. It is also known that thinking about the future is associated with a greater sense of meaning [17]. Thus, negative life events, especially those that may have some potential consequences, can lead to reflection and strengthen sense of meaning. This is consistent with the research indicating that negative events that had consequences for the individual’s future (such as a conflict with another person) were associated with greater meaning [18]. However, everyday experiences are often small and thus easy to overlook, which means that they do not stimulate reflection and thinking of the individual in a way that could contribute to strengthening his/her sense of meaning in life. Hence the proposal to create interventions that would encourage people to seek meaning in mundane events that occur every day [19]. Stress is an inevitable element of our lives, and we experience it not only in the face of traumatic events, but also in response to everyday situations such as interpersonal conflicts or unforeseen events [20]. Therefore, it seems that it would be beneficial to treat such experiences as a potential source of meaning. In our research, we designed a simple intervention that aims to show the participants that daily unpleasant events can also be a source of a sense of meaning, and encourage reflection and drawing conclusions for the future, in order to enhance the sense of meaning in life. Such interventions also fit with the trend toward strengthening mental resilience by focusing on an individual and their constructive responses to a given situation instead of merely protecting them from external threatening factors [21].

Considering one’s life as meaningful has many positive consequences for functioning [14,22] and it is understood as one of the basic elements of well-being [23]. Having a sense of meaning in life is associated with more adaptive ways of coping with stress [24], less anxiety and depression [25], and greater life satisfaction [26]. It is worth noting, however, that there is a difference between having meaning in life and searching for it. Searching for meaning in life, as opposed to having it, is associated with higher levels of anxiety, depression, and negative affect [26]. In recent years, researchers emphasize that we can distinguish two types of well-being: subjective well-being, also referred to in the literature as hedonic well-being, which is related to frequent experiences of positive emotions and pleasure, rare experience of negative affect, and perceiving one’s life as satisfying [27], and psychological well-being, referring to the eudaimonic approach, which focuses on the development and self-realization of the individual and describes life in terms of virtues [28]. Within psychological well-being, Ryff [29] distinguishes six domains: (1) self-acceptance, (2) positive relations with others, (3) autonomy, (4) environmental mastery, (5) purpose in life, and 6) personal growth. It has been shown that there is a positive relationship between meaning in life and psychological well-being, indicating that sense of meaning may be related to self-realization [30].

It seems that one of the factors that is related to both the sense of meaning in life and well-being is self-efficacy [31], though there are still little data available on these relationships. Self-efficacy is a term derived from Albert Bandura’s [32] social learning theory, which defines it as a person’s belief that he or she is able to perform a task. However, researchers point out that self-efficacy also applies to beliefs about the ability to cope with difficulties in new, stressful situations [33]. Beliefs about the ability to deal with various situations are formed based on the experience of the individual. People with a low level of self-efficacy may have difficulty perceiving their life as understandable, and thus have a lower sense of well-being [34]. Researchers point to the mediating role of self-efficacy between meaning in life and well-being; however, previous studies have focused on the hedonic, not the eudemonic, approach [35,36]. Due to the relationships between meaning in life, well-being, and self-efficacy, as well as due to the fact that this study is a designed intervention focused on difficult and stressful everyday events, we decided that it is worth considering self-efficacy in our research.

Due to the fact that the sense of meaning in life changes over the lifetime [37], we decided to focus on strengthening the sense of meaning in life and well-being in emerging adulthood. This stage has been described as one of the most unstable periods in life, accompanied by numerous changes which can be stressful and overwhelming for young people, especially when these changes are perceived as unwanted [38]. Therefore, identifying stressful everyday events as a potential source of meaning could be particularly useful in this age group. What is more, finding meaning in life is one of the developmental tasks of this period [39]. Meaning in life is also a protective factor for mental health, and research has shown that emerging adults are the most vulnerable among all adults to a variety of mental problems [40].

The aim of this study is to explore whether interventions that encourage emerging adults to reflect on trivial stressful events can contribute to a sense of meaning and well-being, and whether there is a relationship between meaning in life, well-being, and self-efficacy. Based on the previously mentioned research, we expect that the respondents participating in the intervention will report an increase in both the sense of meaning in life and well-being. Since previous research concerned mainly subjective well-being [33,34], in our research we want to focus on the relationships between sense of meaning in life, self-efficacy, and psychological well-being.

## 2. Materials and Methods

### 2.1. Study Design and Sampling Procedures

The study was a pretest–posttest control group design. At the beginning, each participant drew, from a stack of white opaque envelopes, a random envelope with a code that assigned them to the intervention group or to the control group. The envelopes were shuffled and their order was random. All data obtained from the participant were labeled with this code.

Each participant came to a meeting with the researcher twice. Participants from the intervention group received specially prepared paper diaries to write down their thoughts at the end of each day for seven days, in accordance with the instructions provided (detailed description of the instructions can be found below). After a week they came to another meeting with the researcher. The control group did not receive any additional tasks and was also asked to come back to see the researcher a week later. The study participants could receive a salary of $9 (some of the respondents resigned from their salary). The results of a priori analysis of statistical power calculated with the GPower calculator for differences between dependent means (matched pairs) with effect size defined as *q* = 0.5 showed that, for error probability set as α = 0.05 and power set as 1 − β = 0.9, the minimum required sample size was 36. The results of a priori analysis of statistical power calculated for linear multiple regression with effect size defined as f^2^ = 0.5 showed that, for error probability set as α = 0.05, power set as 1 − β = 0.9, and number of predictors set as: 2, the minimum required sample size was 30.

The project was approved and financed from the funds earmarked for young scientists and doctoral students at the Faculty of Philosophy of the Jagiellonian University in Krakow. The study was conducted in accordance with the principles of the Declaration of Helsinki. Participation in the research was voluntary, it was possible to resign at any time, and all collected data have been anonymized. The participants received information about the study both orally and in writing and gave their written consent to participate in the study.

### 2.2. Recruitment and Participants

The research was conducted in Krakow (Lesser Poland Voivodeship, Poland) between January and September in 2019 among people in the period of emerging adulthood (between 18 and 29 years old). The study involved 80 young adults (56 women and 24 men). Only those who had not received psychiatric treatment were included in the study. All study participants were students or graduated in the past few years. Young adults who have experienced a traumatic event over the past year (such as the death of a loved one, own or parents’ divorce, or an accident) were excluded from the study because the event could significantly affect their meaning in life. The respondents were invited to participate in the study directly by the researcher as well as by e-mail and by means of advertisements posted on student forums at three universities in Krakow. Over 120 people signed up to participate in the study, but only 85 people came to the second meeting with the researcher on the scheduled date.

### 2.3. Procedure and Measures

During the first meeting, the diaries with instructions, the same for each day, were distributed to persons from the experimental group. Each day, those participants were asked to describe one difficult/stressful situation that took place during this day, the consequences of this situation, what it meant for them, and whether they were able to draw any conclusions from it for the future. The exact instruction contained in the diary was: *Please, think for a moment what your passing day looked like. Think about the fact that everyday difficult and unpleasant situations can also matter in a person’s life. Describe one situation that happened to you today, and then define what consequences it had for you, how important it was for you, and what conclusions you can draw from it for the future.* The significance of a described situation was assessed by the respondents on a scale from 1 to 10 (1—“this situation did not matter to me”; 10—“ this situation was very important to me”). Participants completed their diaries for 7 days. At the first meeting, we explained to the respondents that during the next week we would ask them to write down their observations and reflections at the end of each day, however, we did not say that it could contribute to increasing their sense of meaning or well-being, as this information could affect their later responses in questionnaires. After a week, the participants met with the researcher again. The control group also met the researcher twice to complete the questionnaires, but were not given any additional tasks.

The study used the Meaning in Life Questionnaire [26], the Generalised Self-Efficacy Scale [41], the Ryff Scales of Psychological Well-Being [29,42], and a short questionnaire on demographic variables prepared by the authors of the study. The participants completed each scale twice: during the first meeting with the researcher, and then after a week (except for the short questionnaire on demographic variables).

The Meaning in Life Questionnaire (MLQ) allows measuring the meaning in life on two time planes: present and future. It consists of 10 questions, responded on the 7-point Likert scale (from “absolutely untrue” to “absolute truth”). The Polish version of this tool was used, adapted by Kossakowska, Kwiatek, and Stefaniak [43]. The questionnaire consists of two subscales: the presence of meaning in life and the search for meaning. Cronbach’s alpha index for the subscale measuring the presence of meaning in life was 0.86 and for the subscale used to measure sense-seeking was 0.87.

The Generalised Self-Efficacy Scale (GSE) measures the general beliefs of an individual regarding ability to cope with difficult situations and obstacles. The scale consists of 10 items, to which the individual responds on a 4-point scale (1—“no”; 2—“probably not”; 3—“rather yes”; 4—“yes”). The higher the score on the scale, the greater the individual’s sense of self-efficacy. The Polish version of the scale adapted by Juczyński [44] has satisfactory psychometric properties. Cronbach’s alpha index was 0.85 and the reliability of the scale assessed by the test–retest method was 0.78.

The Ryff Scales of Psychological Well-Being are used to measure mental well-being in the eudaimonistic approach. The research used the Polish adaptation by Karaś and Cieciuch [45], which contains 84 statements reflecting six facets of mental well-being: autonomy, personal development, environmental mastery, life goal, positive relationships with others, and self-acceptance. The individual is to respond to each of the presented statements on a scale from 1 to 6 (1—“I strongly disagree”; 3—“I rather disagree”, 6—“I strongly agree”). It is possible to calculate the score for each subscale as well as the overall score, which consists of the average of all subscales. Cronbach’s alpha index for each subscale was over 0.70.

### 2.4. Statistical Analysis

Means, standard deviations, and measures of normality were calculated for all the measures used in the study. The variables were normally distributed, which allowed for the application of parametric tests. Assumptions for regression analysis, such as the normality of the residual distribution and independence of errors, were met.

To verify whether there were differences in the area of meaning in life, psychological well-being, and self-efficacy between the first (before the intervention) and the second measurement (after the intervention), dependent samples (paired samples) Student’s *t*-tests were carried out. To verify the relationship between meaning in life, psycho-logical well-being, and self-efficacy, the Pearson correlation coefficient was used.

**Figure 1:**
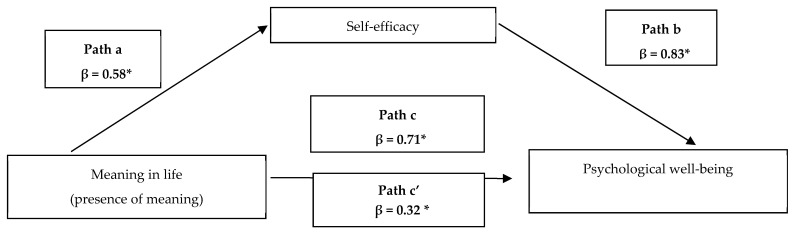
Model of the mediational role of self-efficacy in the relationship between meaning in life and psychological well-being. * *p* < 0.001.; **β**-standardized regression coefficient.

The most popular approach in the psychological literature [46] by Kenny [47] was used to check the mediation effect. This approach involves four steps using multiple regression. At the beginning, the outcome variable is regressed on the predictor to verify whether there is an effect (see Path c in Figure 1). Then, the mediator is regressed on the predictor variable (see Path a in Figure 1). Later, it should be shown that there is a relationship between the mediator and the outcome variable (see Path b in Figure 1). The outcome variable is regressed on both the predictor and the mediator, in the last equation (see Path c’ in Figure 1). If mediation occurs, then after adding the mediator to the model, the strength of the relationship between the predictor and the outcome variable decreases (compare Path c and c’ in Figure 1). To verify whether self-efficacy explains the relationship between the presence of meaning in life and psychological well-being, a mediation analysis was carried out. Due to the fact that the search for meaning in life was not positively correlated with psychological well-being, in our analysis we decided to focus on presence of meaning only. All regression paths were tested following the steps described above.

All the statistical procedures were performed through the use of STATISTICA 13. To check whether mediation takes place, the Sobel calculator was used (http://quantpsy.org/sobel, accessed on: 10 June 2021). For all analyses, the alpha significance level was 5%.

## 3. Results

Descriptive statistics relating to the sociodemographic data of the experimental group and the control group are provided in Table 1. Five cases had to be excluded from the analysis due to incomplete data. Both groups were comparable in terms of age, marital status, place of residence, and not having children. However, there was a gender imbalance, with women dominating the experimental group.

Significant differences were noted in psychological well-being, especially in the area of relationships with others and purpose in life, as well as self-efficacy. There were no differences in the level of meaning in life (Table 2). There were no differences between the first and second measurement in any of the areas in the control group (without intervention) (Table 3).

The analysis of the data showed a relationship between meaning in life, psychological well-being, and self-efficacy. The overall score on the meaning in life was positively correlated with psychological well-being (r = 0.51; *p* < 0.001) and self-efficacy (r = 0.35; *p* < 0.01). The positive relationship between psychological well-being and self-efficacy (r = 0.83; *p* < 0.001) was also revealed. Positive correlations were also found between the presence of meaning in life and all six domains of psychological well-being, and they were particularly strong in the case of the subscales: environmental mastery (r = 0.65; *p* < 0.001) and purpose in life (r = 0.86; *p* < 0.001). There was no relationship between search for meaning in life and psychological well-being or self-efficacy. Furthermore, self-efficacy was correlated with all subscales of psychological well-being, especially to self-acceptance (r = 0.70; *p* < 0.001) and environmental mastery (r = 0.77; *p* < 0.001). There was also a positive relationship between the assessment of the importance of the event and meaning in life (r = 0.53; *p* < 0.01).

Multiple regression analysis was performed to explore relationship between sense of meaning in life, self-efficacy, and psychological well-being (Table 4). In Step 1, presence of meaning in life significantly determined psychological well-being (β = 0.70; *p* < 0.001). In Step 2, presence of meaning in life made it possible to significantly predict self-efficacy (β = 0.58; *p* < 0.001). To check whether mediation takes place, the Sobel calculator was used. The Aroian test result was statistically significant (z = 4.48; SE = 0.11; *p* = 0.0007). In Step 3, to check the nature of this mediation, a multivariate regression analysis was performed, where the dependent variable was psychological well-being, and presence of meaning in life and self efficacy were predictors. Both variables made it possible to predict the level of psychological well-being in a significant way. Presence of meaning in life predicted the level of psychological well-being (β = 0.32; *p* < 0.001) to a lesser extent than in the situation without the presence of a mediator (from β = 0.71 to β = 0.32; *p* < 0.001). Thus, it was found that partial mediation takes place. The mediation model is shown in Figure 1.

Presence of meaning had a direct positive effect on self-efficacy (Path a), and self-efficacy had a direct impact on psychological well-being (Path b). When self-efficacy was introduced into the model, both presence of meaning in life and self-efficacy had an impact on psychological well-being; however, the predictive effects of presence of meaning for psychological well-being decreased (Path c’). Indirect influence of presence of meaning in life on psychological well-being through self-efficacy was observed.

The content of diaries filled out by participants from the experimental group was also analyzed. Participants’ experiences were open-coded, looking for common themes. Among the everyday stressful situations described by the respondents, it was possible to distinguish three main categories. The first category concerned interpersonal conflicts, such as quarrels with a partner, misunderstandings between family members or friends, or disagreements between roommates. Seventy-five percent of the described events were related to this category. The second category included situations describing problems with achieving the set goals and implementation of plans (e.g., insufficient preparation for the exam due to poor time management; unpreparedness for classes; failure to perform certain activities systematically despite strong resolve). Twenty percent of the described situations were assigned to this category. The remaining 5% were situations related to random accidents (e.g., car crash, wallet loss, forgetting the keys to the apartment).

## 4. Discussion

Our research shows that intervention encouraging reflection on everyday stressful events may strengthen the sense of psychological well-being in the area of positive relationship with others and purpose in life among emerging adults. It may be related to the events that the respondents thought about. Seventy-five percent of the described events were related to interpersonal problems with romantic partners, family members, or friends, and 20% of problems with achieving the goals set for themselves. Since the respondents primarily focused on drawing conclusions for the future from situations related to interpersonal relationships, it seems logical that we can observe significant changes in this area. Such a selection of events may also be related to the development period of the participants. One of the main tasks of early adulthood is developing close intimate relationships [38,48]; hence, these experiences may be of particular importance for this age group. Previous research has shown that insight is a statistically significant predictor of psychological well-being [49]. Reflecting on the consequences of one’s own actions and drawing conclusions seem to promote greater clarity and understanding of one’s thoughts and behaviors, which are the essence of insight [50], and thus contribute to an increase in psychological well-being. Importantly, people in the experimental group were not informed before the end of the study that reflection on everyday events may strengthen their sense of meaning in life or well-being, which allows us to assume that the observed change is the result of intervention. What is interesting is that we also noted a change in the field of self-efficacy. Perhaps reflecting on one’s own behavior and on how to behave in a similar situation in the future strengthens the belief in the possibility of coping with difficulties. Since intervention focused on reflection on everyday stressful events can, to some extent, foster the well-being of individuals, it may be of great importance in both preventive and therapeutic programs. The tested intervention is very simple, does not require large financial outlays, and can also be implemented without constant supervision of a specialist. It is possible that this type of reflection on everyday stressful events could be used not only in the form of written-down thoughts, but also as a subject of conversation with a counselor, coach, or therapist. It is known that meaning-centered interventions among emerging adults work well, for example, in the context of professional career [51]. Research has also shown that meaning-centered interventions are helpful in treating anxiety and mood disorders [52], can reduce stress, and contribute to the improvement of the quality of life. It is postulated that such interventions should be more accessible, especially to people in transitional moments in life [53], such as transition from adolescence to adulthood. Considering that finding meaning in life is one of the developmental tasks of this period [39], identifying everyday events as potential sources of meaning may be helpful. No significant changes in meaning in life were observed, which may be related to both the short intervention time and the tool used to measure meaning in life. The Meaning in Life Questionnaire (MLQ) measures the global sense of meaning in life, which is rather stable [54]. It differs from daily meaning which fluctuates and can change day to day [55,56]. It seems, therefore, that with such intervention planned, a better solution would be to measure daily meaning in life as this would more accurately capture possible changes.

Our research shows a positive relationship between meaning in life and psychological well-being, which is consistent with the results obtained previously by other researchers [57,58], and between meaning in life and self-efficacy. As expected, positive correlations concerned total result of meaning in life, as well as presence of meaning, while searching for meaning correlated neither with psychological well-being nor with self-efficacy. There was also positive relationship between self-efficacy and well-being which confirms the results of previous studies [59]. Knowledge of this relationship between meaning in life, self-efficacy, and psychological well-being, can be useful for interveners, psychologists, and coaches who, while working with clients and patients, reflect on how to strengthen their sense of well-being. It seems that including a sense of meaning in life and self-efficacy in such interventions could bring more benefits, especially since both help to build resilience [60,61], which is a valuable resource of the individual and is of great significance for coping with difficult situations in life. Our research was conducted before the outbreak of the COVID-19 pandemic, however, it seems that in the face of the lockdown and the forced various changes in the daily functioning of individuals, building resilience became even more important [62].

Researchers noted a mediating role of self-efficacy between meaning in life and subjective well-being [35,36]. We wanted to check whether we would be able to observe the same relationship in the case of psychological well-being. To our knowledge, this is the first study to examine this type of relationship. The conducted analysis showed that self-efficacy is a mediator between meaning in life and psychological well-being. This result deepens the knowledge about meaning in life, self-efficacy, and psychological well-being, and at the same time shows the complexity of the relationship between these three constructs, which is a good basis for further research in this area. It seems that it would be worth taking a closer look at the relationship between these constructs, especially since a single report from research on cardiac patients has recently appeared, in which the mediating role of meaning in life between self-efficacy and psychological well-being was pointed out [63].

### Limitations and Future Directions

It is worth noting that our research was one of the first attempts to strengthen the sense of meaning and psychological well-being through an intervention encouraging reflection on stressful everyday events. The obtained results indicate that this type of intervention has potential; however, further research in this area is certainly necessary, particularly randomized controlled trials that could provide more reliable results. Firstly, the experimental group was somewhat small and quite homogeneous—it consisted only of people in emerging adulthood who had higher education or were university students. Participants also did not have any children and, as research has shown, having children may contribute to sense of meaning in life [64]. However, it is worth emphasizing that the fact that the participants were not married and did not have children is typical for emerging adulthood. Arnett [38] pointed out that, nowadays, young adults postpone making important life decisions, such as getting married or starting a family. We are aware that not all emerging adults go to university and receive higher education, and, therefore, in future research, it would be worthwhile to advertise them not only on student forums, but also on social media used by young adults. Therefore, it would be worth checking the effectiveness of such interventions on people of different ages, with different levels of education and a different life situation. Another limitation of the study concerns the fact that the majority of participants were women and, in the experimental group, there were almost two times less men than in the control group. It cannot be ruled out that the effectiveness of the intervention may be related to gender. Due to the disproportion between men and women, we did not make inter-gender comparisons, and this is one of the elements that should be taken into account in future studies. A relatively small number of people volunteered for participation in the study (and some of them did not attend the second meeting with the researcher), therefore it cannot be ruled out that the people taking part in the study differed in some features from the rest of the population of emerging adults. The intervention was relatively short, which may have resulted in the inability to observe the desired change in the sense of meaning in life. We propose that the intervention should last at least two weeks, as in the case of gratitude interventions, which are to strengthen the sense of well-being [65,66]. It also seems that future research should provide a more precise instruction that would indicate the need for reflection in various areas of an individual’s functioning. As mentioned above, it would also be worth considering measuring daily meaning, instead of global meaning, as it is more sensitive to change. However, it is not certain that prolonging the intervention itself would bring greater benefits to the people participating in it, therefore it would also require further research. It would also be important to determine how long the positive effects of the intervention last, which unfortunately was not included in our research. Due to the fact that there is practically no data on the mediating self-efficacy effect between meaning in life and psychological well-being, this is certainly an area that requires further exploration.

## 5. Conclusions

Based on the data collected and analyzed in our study, the following conclusions can be drawn:

1. Reflection on everyday stressful events has the potential to enhance psychological well-being among emerging adults, especially in the area of positive relationships with others;
2. There is a positive association between the presence of meaning in life, but not search of meaning, self-efficacy, and well-being.
3. Meaning in life and self-efficacy are predictors of psychological well-being. What is more, self-efficacy is a mediator between presence of meaning in life and psychological well-being;
4. Both meaning in life and self-efficacy should be considered in planning interventions to foster psychological well-being.

## Figures and Tables

**Table 1:** Characteristics of the sample (*n* = 80).

	Group with Intervention		Control Group	
	M	SD	M	SD
Age	20.78	1.35	21.25	2.29
	*n*	%	*n*	%
Sex				
Male	9	22.5	15	37.5
Female	31	77.5	23	62.5
Place of residence				
Town	28	69	35	87.5
Village	12	31	5	12.5
Marital status				
Single	21	52	17	43
Informal relationship	19	48	23	57
Married	0	0	0	0
Children				
Yes	0	0	0	0
No	40	100	40	100
Job				
Yes	7	17	14	36
No	33	83	26	64

M (mean), SD (standard deviation).

**Table 2:** Comparison of the level of meaning in life, psychological well-being, and self-efficacy before and after intervention in experimental group (with intervention) (*n* = 40).

	Before Intervention		After Intervention		t	*p*
	M	SD	M	SD		
**Meaning in life** Total result	46.64	7.81	46.59	10.92	−0.14	0.88
Presence of meaning in life	20.21	6.29	21.09	7.51	−1.83	0.07
Search of meaning in life	26.24	3.77	25.48	5.39	1.01	0.31
**Psychological well-being** Total result	55.67 *	9.93	59.22 *	9.79	−2.25 *	0.02 *
Self-acceptance	52.97	16.55	53.34	15.44	−0.51	0.60
Positive relationships with others	59.34 ***	8.32	65.07 ***	8.77	−11.40 ***	0.001 ***
Autonomy	56.41	13.76	57.51	13.36	−0.93	0.35
Environmental mastery	56.78	12.20	55.08	13.68	1.16	0.25
Purpose in life	54.85 **	9.83	58.21 **	11.91	−3.15 **	0.003 **
Personal growth	64.14	8.99	65.41	7.70	−1.87	0.06
**Self—efficacy** Total result	28.06 **	5.95	29.60 *	5.91	−2.82 **	0.007 **

* Statistically significant results; * *p* ≤ 0.05; ** *p* ≤ 0.01; *** *p* ≤ 0.001; M (mean), SD (standard deviation), t (t-statistic), *p* (*p*-value).

**Table 3:** Comparison of the level of meaning in life, psychological well-being, and self-efficacy before (pretest) and after a week (posttest) in control group (without intervention) (*n* = 40).

	Pretest		Posttest		t	*p*
	M	SD	M	SD		
**Meaning in life** Total result	47.50	5.56	47.75	6.03	−1.74	0.10
Presence of meaning in life	22.12	3.98	22.25	3.95	−0.11	0.91
Search of meaning in life	25.37	4.52	26.50	3.89	−1.88	0.07
**Psychological well-being** Total result	60.31	3.64	60.64	4.62	−0.64	0.52
Self-acceptance	61.37	4.03	62.38	5.48	−0.67	0.50
Positive relationships with others	59.12	9.05	58.75	4.84	0.28	0.78
Autonomy	58.87	13.24	57.87	12.37	0.99	0.33
Environmental mastery	60.00	5.77	59.37	4.86	0.67	0.50
Purpose in life	57.25	5.90	59.62	7.62	−3.88	0.08
Personal growth	65.25	4.72	62.37	7.29	1.58	0.13
**Self—efficacy** Total result	29.25	3.29	29.87	3.98	−1.03	0.31

M (mean), SD (standard deviation), t (t-statistic), *p* (*p*-value).

**Table 4:** Multiple regressions analysis on the mediating effects of self-efficacy (n = 80).

	Outcome Variable	Predictor Variable	β	SE β	B	*p*	R^2^
Step 1 (path c)	Psychological well-being	Presence of meaning in life	0.71	0.10	0.93	<0.001	0.52
Step 2 (path a)	Self-efficacy	Presence of meaning in life	0.58	0.11	0.50	<0.001	0.39
Step 3 (path c’)	Psychological well-being	Presence of meaning in life	0.32	0.09	1.02	<0.001	
		Self-efficacy	0.62	0.09	0.42	<0.001	0.76

β (standardized regression coefficient), SE β (standard error for the standardized beta), B (unstandardized regression coefficient), *p* (*p*-value), R^2^ (coefficient of determination).

## Data Availability

The data that support the findings of this study are available from the corresponding author, upon reasonable request.
